# Combining Auxin-Induced Degradation and RNAi Screening Identifies Novel Genes Involved in Lipid Bilayer Stress Sensing in *Caenorhabditis elegans*

**DOI:** 10.1534/g3.120.401635

**Published:** 2020-09-21

**Authors:** Richard Venz, Anastasiia Korosteleva, Elisabeth Jongsma, Collin Y. Ewald

**Affiliations:** Eidgenössische Technische Hochschule Zürich, Department of Health Sciences and Technology, Institute of Translational Medicine, Schwerzenbach-Zürich CH-8603, Switzerland

**Keywords:** Lipid bilayer stress, Lipotoxicity, Unfolded Protein Stress, Auxin-induced degradation, CREB3, NRF2, MDT-15

## Abstract

Alteration of the lipid composition of biological membranes interferes with their function and can cause tissue damage by triggering apoptosis. Upon lipid bilayer stress, the endoplasmic reticulum mounts a stress response similar to the unfolded protein response. However, only a few genes are known to regulate lipid bilayer stress. We performed a suppressor screen that combined the auxin-inducible degradation (AID) system with conventional RNAi in *C. elegans* to identify members of the lipid bilayer stress response. AID-mediated degradation of the mediator MDT-15, a protein required for the upregulation of fatty acid desaturases, induced the activation of lipid bilayer stress-sensitive reporters. We screened through most *C. elegans* kinases and transcription factors by feeding RNAi. We discovered nine genes that suppressed the lipid bilayer stress response in *C. elegans*. These suppressor genes included *drl-1*/MAP3K3, *gsk-3*/GSK3, *let-607*/CREB3, *ire-1*/IRE1, and *skn-1*/NRF1,2,3. Our candidate suppressor genes suggest a network of transcription factors and the integration of multiple tissues for a centralized lipotoxicity response in the intestine. Thus, we demonstrated proof-of-concept for combining AID and RNAi as a new screening strategy and identified eight conserved genes that had not previously been implicated in the lipid bilayer stress response.

Biological membranes play an important role in protein folding, signaling, secretion, and the turnover of proteins. Changes in the lipid composition of a membrane alter its properties, and thus, interferes with its function and leads to lipid bilayer stress (LBS) (Covino *et al.* 2018). Maintaining the membranes’ composition is, therefore, crucial for a cell. High dietary intake of saturated fatty acids leads to a metabolic syndrome referred to as lipotoxicity (Ertunc and Hotamisligil 2016). On a cellular level, elevated levels of saturated fatty acids alter membrane composition. Sensitive for these changes is the endoplasmic reticulum (ER), which is a significant site for protein and lipid synthesis, and the main site of intracellular calcium storage (Schwarz and Blower 2016). Lipid disequilibrium interferes with secretory capacity and renders cells specialized in secretion, such as insulin-producing beta cells, susceptible to cell death (Preston *et al.* 2009; Acosta-Montaño and García-González 2018). Although LBS has been suggested to play a major part in disease progression, the spectrum of the underlying molecular players sensing LBS remains to be identified.

The unfolded protein response (UPR) sensors IRE1, PERK1, and ATF6 are sensitive to changes in membrane fluidity (Koh *et al.* 2018). On the molecular level, IRE1, PERK1, and ATF6 act in parallel in response to unfolded proteins (Figure 1a). Activated IRE1 splices *XBP1* mRNA to stabilize the transcript and allow translation of the spliced XBP1 transcription factor (Figure 1a). PERK1 phosphorylates the initiation factor eIF2alpha, which reduces translation rate and allows preferential translation of genes containing upstream open reading frames (uORFs), such as the transcription factor ATF4 (Harding *et al.* 2000) (Figure 1a). During ER stress, ATF6 translocates from the ER to the Golgi, where it is cleaved by proteases termed S1P and S2P. Cleaved ATF6 migrates to the nucleus and acts as a transcription factor (Figure 1a). These transcription factors co-regulate many targets, but how the downstream targets of the three arms of the UPR restore membrane homeostasis in detail remains unknown.

**Figure 1:**
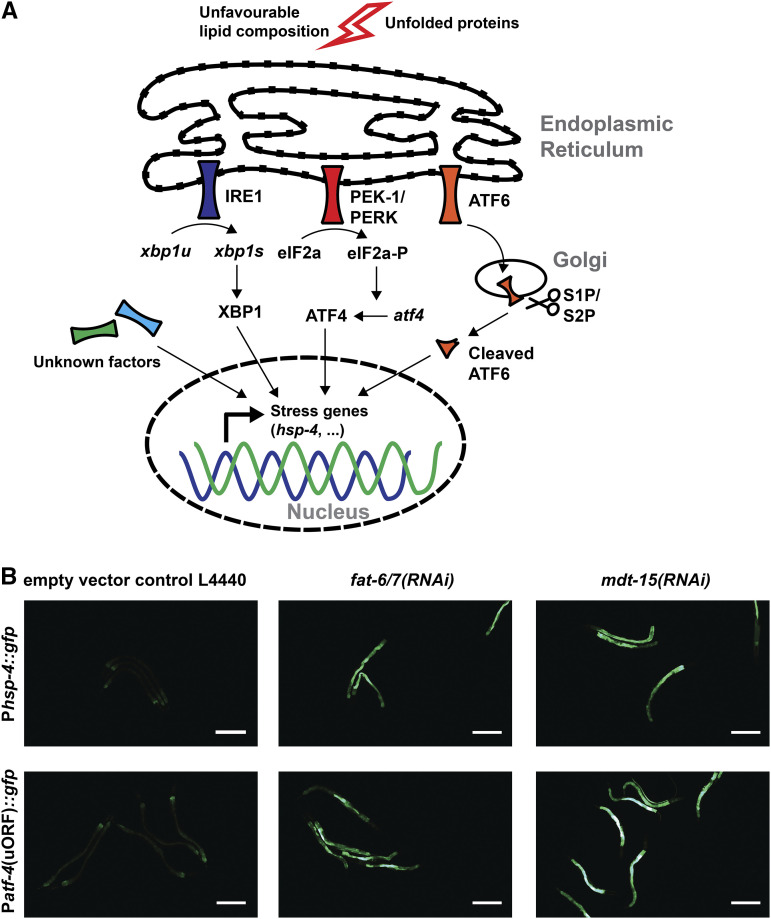
Integrated stress response of *C. elegans* a) Model of the unfolded protein and lipid bilayer stress response. b) P*hsp-4*::*gfp* and P*atf-4*(uORF)::gfp are activated by *fat-6*/*7**(RNAi)* and *mdt-15**(RNAi)*. Scale bar = 200 μm.

In *C. elegans*, loss of fatty acid desaturases *fat-6* and *fat-7* or *mdt-15*, which is a mediator subunit required for *fat-6*/*7* expression, leads to a higher ratio of saturated fatty acids in the membrane. This activates the ER stress reporter *hsp-4*::*gfp* via the IRE-1/XBP-1 axis (Hou *et al.* 2014). Supplementing C18:1n-9 fatty acid oleate, the product of the FAT-6/*7* stearoyl-CoA-desaturases, either partially rescued, in the case of *mdt-15* knockdown, or entirely rescued, when *fat-6*/*7* knocked down the induction of ER stress reporter *hsp-4*::*gfp* (Hou *et al.* 2014). The partial rescue of the *mdt-15* knockdown may be via other processes or functions that are disrupted in these animals leading to ER stress. The mediator subunit *mdt-15* regulates lipid metabolism and is also involved in many other processes, including immunity, stress defense, detoxification, and mitochondrial stress (Taubert *et al.* 2008; Mao *et al.* 2019; Lee *et al.* 2019). Also, activation of the ER stress sensor can also be achieved by depleting the cell’s phosphatidylcholine levels, for example, via knockdown of *sams-1*/MAT1A, s-adenosyl methionine synthetase (Hou *et al.* 2014). Curiously, the signature of lipid bilayer stress response is different from the canonical UPR in *C. elegans* (Hou *et al.* 2014; Koh *et al.* 2018). This argues for an additional layer of regulation that fine-tunes the output during activation of the three UPR arms (Figure 1a).

Genetic mutant screening for members of the UPR has been successful (Calfon *et al.* 2002). However, setting up genetic screens with essential genes that either cause lethality or developmental defects are difficult. RNAi-based forward screens can bypass genes that cause embryonal lethality or developmental defects. However, feeding more than one RNAi simultaneously was previously reported to produce poor results (Min *et al.* 2010). This suggests a bottleneck for screening strategies where one would like to screen for suppressors of a phenotype caused by a knockdown using an RNAi-mediated screen.

The auxin-inducible degradation (AID) system has been recently introduced to mediate fast and reversible degradation of targeted proteins in *C. elegans* (Zhang *et al.* 2015). A protein of interest can be tagged with a short 68 amino acid sequence (degron), which is recognized by the E3 ubiquitin ligase TIR1, derived from *Arabidopsis thaliana*, in the presence of a small molecule called auxin (Zhang *et al.* 2015; Martinez *et al.* 2020). Ubiquitination targets the degron-tagged protein for fast degradation by the proteasome. Depletion times of less than 30 min have been reported for cytosolic proteins after transferring *C. elegans* co-expressing a degron-tagged protein and TIR1 on plates containing auxin (Zhang *et al.* 2015). The AID is, therefore, faster and more efficient than RNAi. Since AID initiates protein degradation and RNAi initiates mRNA degradation, these two systems do not compete with each other and can be used in parallel.

Here, we identify suppressors of lipid bilayer stress response using a novel approach combining AID and RNAi-based forward genetic screening. Degradation of MDT-15 by AID was used to induce LBS, which was visualized using the ER-stress reporters P*atf-4*(uORF)::gfp and P*hsp-4*::*gfp*. We screened RNAi libraries targeting kinases and transcription factors. Out of 868 genes, we identified one known and eight novel hits that robustly blocked LBS response upon MDT-15 degradation.

## Materials and Methods

### C. elegans strains

All strains were maintained at 20° on OP50 *Escherichia coli*, as described (Stiernagle 2006).

IJ1729: *ieSi57* [P*eft-3*::TIR1::mRuby::*unc-54* 3′UTR; *cb-unc-119*] II; *yh44* [*mdt-15*::degron::EmGFP] III. (Lee *et al.* 2019), SJ4005: *zcIs4* [P*hsp-4*::GFP] V. (Harding *et al.* 2000), LD1499: [P*atf-4*(uORF)::GFP::*unc-54*(3′UTR)], LSD2096: *ieSi57* [P*eft-3*::TIR1::mRuby::*unc-54*(3′UTR); *cb-unc-119*] II; *yh44* [*mdt-15*::degron::EmGFP] III; [P*atf-4*(uORF)::GFP::*unc-54*(3′UTR)], LSD2102: *ieSi57* [P*eft-3*::TIR1::mRuby::*unc-54* 3′UTR; *cb-unc-119*] II; *yh44* [*mdt-15*::degron::EmGFP] III; *zcIs4* [P*hsp-4*::GFP] V.

**Figure 2:**
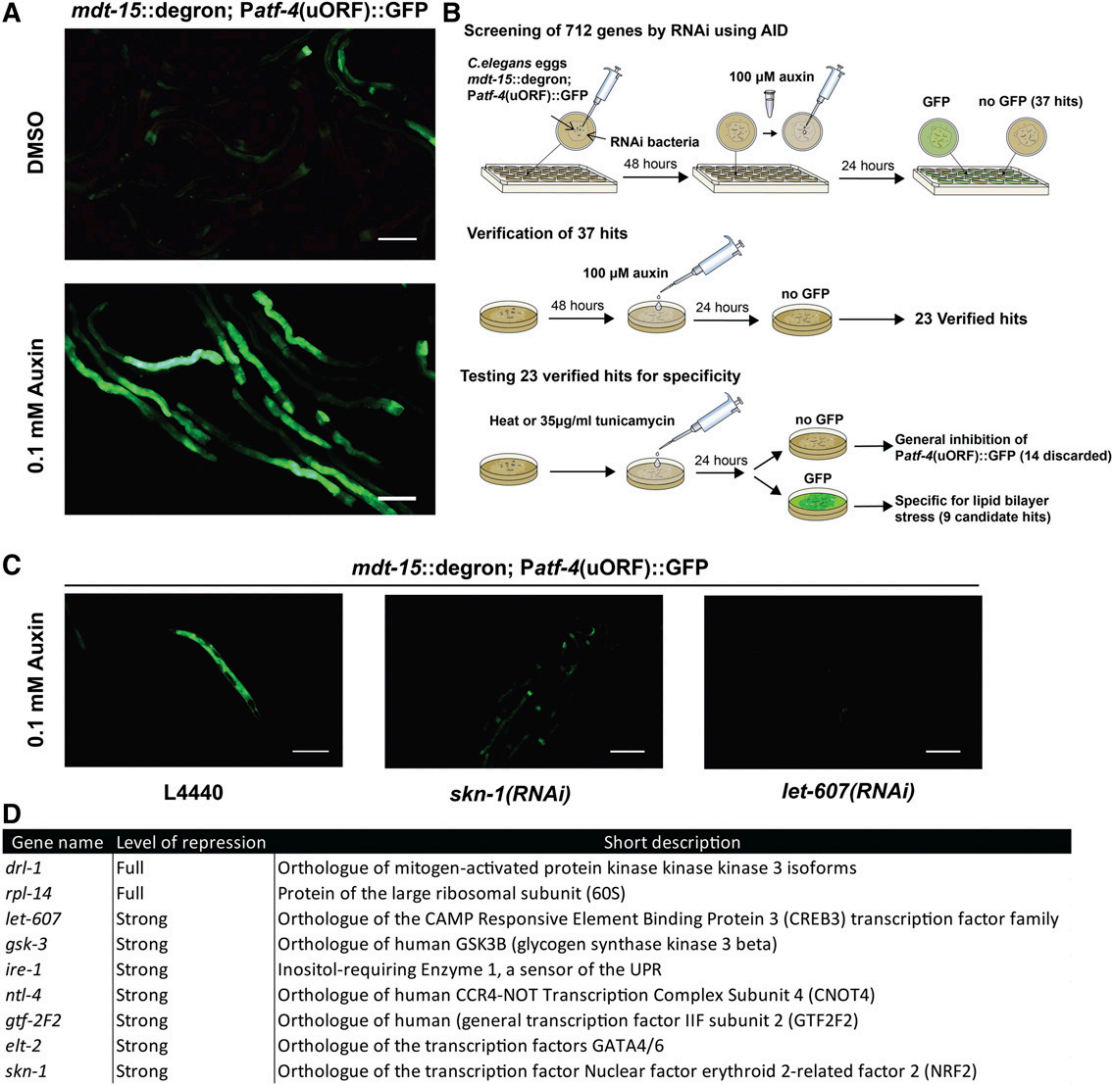
Suppressor screen of LBS a) Addition of 0.1 mM auxin degrades *mdt-15*::degron and leads to expression of P*atf-4*(uORF)::gfp in LSD2096. Pictures were taken 24 h after auxin addition. Scale bar = 200 μm b) Summary of the screening outline. c) Treatment with 0.1 mM auxin to degrade *mdt-15*::degron after *skn-1* and *let-60*7 RNAi represses activation of P*atf-4*(uORF)::gfp in LSD2096. Pictures were taken 24 h after auxin treatment. Scale bar = 200 μm.

The screening strain was generated by crossing IJ1729 males with LD1499. 48 F2s were singled out and their offspring were placed onto plates containing 100 μM auxin and the upregulation of the reporter was determined (Figure 2a). In parallel, IJ1729 was crossed to SJ4005.

### Microscopy

For image acquisition, the animals were placed onto fresh 2% agar pads and anesthetized with 1 mM tetramisole, as previously described (Teuscher and Ewald 2018). Images were obtained using an upright bright field fluorescence microscope (Tritech Research, model BX-51-F) with an attached camera (model DFK 23UX236).

### Quantification of GFP fluorescence

GFP fluorescent levels were scored by inspecting transgenic animals with a fluorescent dissecting scope while still on the culturing/treatment condition plates (Ewald *et al.* 2017). GFP intensity was scored and categorized: 0= none or very low GFP usually corresponding to the untreated control, 1= low, 2= medium, and 3= high GFP fluorescent signal.

### Preparation of auxin

70 mg of auxin (3-Indoleacetic acid, Sigma #I3750) was dissolved in 10 mL DMSO to yield a 40 mM stock solution and stored at 4°. The stock was further diluted in M9 to 100 μM before use.

### Suppressor screen design

A detailed step-by-step protocol can be found in the supplementary, and a schematic outline is shown in Figure 2b.

Briefly, 24-well plates were filled with Nematode Growth Medium (NGM) containing ampicillin (100 μg/ml), tetracycline (12.5 μg/ml), and 1mM Isopropyl β-d-1-thiogalactopyranoside (IPTG) seeded with 50 μl of freshly grown RNAi bacteria and dried in a sterile laminar flow cabinet. The following day, plates containing gravid LSD2096 adults were washed off and discarded, and the laid eggs were scratched off and collected. Approximately 30-40 eggs were pipetted into each well and incubated at 20°. After 48 hr, the wells were top coated with 50 μl of 100 μM auxin and dried in a sterile laminar flow cabinet at 20° overnight. The following day, the wells were screened for the suppression of the GFP signal. The kinase and transcription factor libraries were screened twice. The preliminary hits had to pass three additional runs on 6 cm plates successfully.

### Single and double knockdown by RNA interference for candidate gene validation for LBS

RNAi and double RNAi were performed as described (Ewald *et al.* 2017).

#### For single RNAi:

RNAi bacteria cultures were grown overnight in Lysogeny Broth (LB) medium with carbenicillin (100 µg/mL) and tetracycline [12.5 µg/mL], diluted and grown again for 4-6 hr. Bacteria were then concentrated by centrifugation and induced with 1 mM IPTG and spread onto NGM plates containing 1 mM IPTG, tetracycline (12.5 µg/mL) and ampicillin (50 µg/mL). For the empty RNAi vector (EV), plasmid pL4440 was used as a control.

#### For double RNAi:

bacterial RNAi cultures were grown separately, and after the concentration step, mixed in a 1:1 ratio and seeded onto culture plates, as described above for single RNAi.

### Heat-shock and tunicamycin treatment

Animals, RNAi bacteria, and plates were prepared as above, without the addition of auxin. Heat-shock was carried out for 1 hr at 37°, incubated for 5 hr at 25°, and GFP expression was determined in the animals. Plates were top coated with 0.5 ml of 35 μg/ml tunicamycin (Sigma, T7765), incubated for 6 hr at 25°, and GFP expression was again determined.

### Data availability

Strains used in this study are either available from CAENORHABDITIS GENETICS CENTER (CGC) or upon request. Supplementary materials are available at figshare: https://doi.org/10.25387/g3.12783044.

## Results

Lipid bilayer stress can be induced by knocking down *mdt-15* or *fat-6*/*7*, resulting in the upregulation of ER-stress reporter P*hsp-4*::*gfp* (Hou *et al.* 2014). We confirmed the induction of P*hsp-4*::*gfp* upon RNAi against *mdt-15* and *fat-6*/*7* (Figure 1b). We tested a second ER-stress reporter P*atf-4*(uORF)::gfp, which was also induced upon knockdown of *mdt-15* or *fat-6*/*7* RNAi (Figure 1b). The P*atf-4*(uORF)::gfp reporter contains two upstream open reading frames (uORF) in the 5′ untranslated region (Rousakis *et al.* 2013). Similar to mammalian ATF4, under unstressed conditions, *atf-4* mRNA is not translated but under conditions that lead to a global reduction of protein synthesis (Rousakis *et al.* 2013; Young and Wek 2016). For screening purposes, we preferred P*atf-4*(uORF)::gfp over P*hsp-4*::*gfp* for its stronger induction of GFP, allowing easier detection in 24- or 96-well plates. Crossing *mdt-15**(**tm2182**)* mutant with P*atf-4*(uORF)::gfp led to heterogeneous GFP expression; therefore, it was difficult to use this strain for screening. Hence, we switched to an endogenously degron-tagged *mdt-15* strain (Lee *et al.* 2019). Unstressed MDT-15::degron *C. elegans* expressed P*atf-4*(uORF)::gfp only at basal levels at 20°. Incubation with 100 μM auxin for 24 hr increased GFP levels drastically and homogenously throughout the *mdt-15*::*degron*; TIR1; P*atf-4*(uORF)::gfp transgenic animals (Figure 2a), but did not induce GFP fluorescence in wild-type P*atf-4*(uORF)::gfp (Supplementary Figure 1a, Data Source File 1). Upon treatment with auxin, we also observed typical *mdt-15* phenotypes, such as small body size, reduced brood size, and a pale appearance in *mdt-15*::*degron*; TIR1; P*atf-4*(uORF)::gfp animals, consistent with previous reports (Lee *et al.* 2019). An additional phenotype was observed in our screening strain, the eggs of untreated *mdt-15*::*degron*; TIR1; P*atf-4*(uORF)::gfp animals were sensitive to bleaching, normally used to synchronize *C. elegans* populations. Either, our *mdt-15*::*degron*; TIR1; P*atf-4*(uORF)::gfp screening strain carried a background mutation or degron-tagged *mdt-15* may be partially hypomorph. However, the untreated *mdt-15*::*degron*; TIR1; P*atf-4*(uORF)::gfp screening strain appeared superficially wild type without any induction of GFP. We did not outcross the strain but rather decided to avoid bleach synchronization and continued with our screen by collecting laid eggs off the bacterial lawn. Thus, we established the *mdt-15*::*degron*; TIR1; P*atf-4*(uORF)::gfp strain for screening for LBS suppressors.

Further insight into LBS was gained by taking a targeted RNAi approach. We decided to screen through the majority of *C. elegans* kinases (382 out of the 438 kinases; (Lehmann *et al.* 2013)) and about one-third of all transcription factors (330 out of 934 genes; (Reece-Hoyes *et al.* 2005; Kim *et al.* 2018)) (Data Source File 1). Our first-pass screening round of the total 712 genes (Data Source File 1) resulted in 6 kinases and 31 transcription factors (Figure 2b). To sort out false positives, we tested the preliminary hits on 6 cm plates, which resulted in 23 verified hits that blocked P*atf-4*(uORF)::gfp induction upon *mdt-15* degradation (Figure 2b, 2c; Supplementary Table 1). To investigate whether these 23 hits were specific for lipid bilayer stress, and not general inhibitors of the unfolded protein response, we heat-shocked the animals and treated them with the N-glycosylation-inhibitor tunicamycin (Figure 2b). Out of the 23 hits, we identified 11 hits specific to LBS. The majority of clones that did not pass this step were positive controls of GFP RNAi from the screening libraries (Supplementary Table 1). Reassuringly, we detected *xbp-1*, a transcription factor spliced by IRE-1 (Supplementary Table 1). XPB-1 is known to upregulate *hsp-4* mRNA during UPR (Calfon *et al.* 2002). To rule out transgene-specific effects, we crossed P*hsp-4*::*gfp* into *mdt-15*::*degron*;TIR1 and tested the hits that had passed the previous steps (Figure 2b). Only the weakest hit, *ztf-1*, did not pass this step (Supplementary Table 1). In addition, knockdown of *xbp-1* resulted in the complete absence of any GFP expression even in the control P*atf-4*(uORF)::gfp strain, suggesting that the transcription factor XBP-1 may be necessary for general expression of *atf-4* in *C. elegans*. Together with XBP-1s importance in transcribing *hsp-4*, it is difficult to argue whether *xbp-1* is necessary for LBS, and further research is required to elucidate this. Thus, we ended up with nine candidate suppressors of LBS from our combined AID with RNAi screen (Figure 2d).

To validate that the nine potential candidates were suppressors of LBS and were not only specific to *mdt-15* degradation, we performed single and double RNAi treatment either in combination with *fat-6*/*7**(RNAi)* or *sams-1**(RNAi)*. We found that single knockdown of the candidate genes did not induce P*atf-4*(uORF)::gfp reporter, except for RNAi against *ire-1* or *let-607* which induced the P*atf-4*(uORF)::gfp reporter (Supplementary Figure 1b), suggesting that loss of either these latter two genes may cause ER stress. By contrast, knockdown of *ire-1* or *let-607* suppressed P*atf-4*(uORF)::gfp induction upon *mdt-15* degradation with 0.1 mM auxin (Figure 2) or with a higher concentration of 1 mM auxin (Supplementary Figure 1c-g). Reassuringly, double RNAi of *fat-6*/*7* with *ire-1* or *let-607* and double RNAi of *sams-1* with *ire-1* or *let-607* displayed strong suppression of the P*atf-4*(uORF)::gfp reporter (Supplementary Figure 2; Data Source File 1). Similarly, double RNAi of candidate genes with either *fat-6**/7* or *sams-1* exhibited full suppression of P*atf-4*(uORF)::gfp reporter, except for *elt-2* knockdown (Supplementary Figure 2). Knockdown of *elt-2* did not suppress *fat-6*/*7**(RNAi)* (Supplementary Figure 2a), but suppressed *sams-1**(RNAi)* P*atf-4*(uORF)::gfp reporter (Supplementary Figure 2b). This suggests a differential genetic activation of LBS from a higher ratio of saturated fatty acids in the membranes compared to depleting the cell’s phosphatidylcholine levels. Thus, our screening approach identified nine high confidence candidates. Eight of these nine candidates have not previously been described to mediate LBS in *C. elegans*.

## Discussion

**Figure 3:**
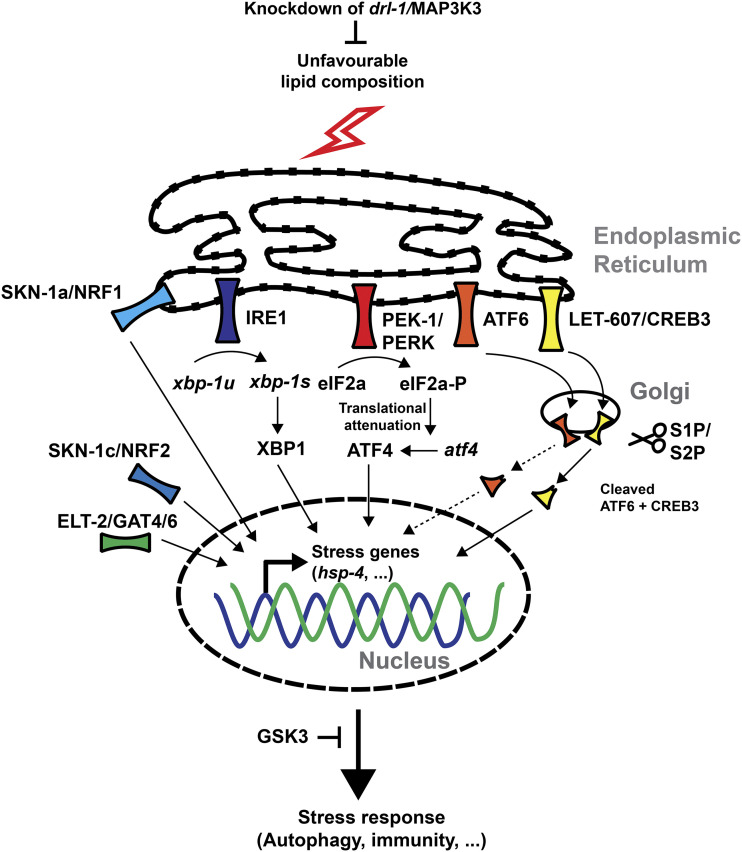
Hypothetical model of LBS in *C. elegans* The updated model of LBS in *C. elegans* indicates a complex network of transcription factors and up- and downstream modulators. 6 out of 9 of our hits are included in this model.

Here we report the first screening approach combining auxin-induced degradation with RNA interference. With our novel approach of combining AID and RNAi screening, we were able to bypass developmental and lethal obstacles caused by the depletion of *mdt-15*. Our screen revealed a known molecular player (IRE-1) and identified several new genes important to mount a proper LBS response. Thus, our results provide proof-of-concept and support the feasibility of combined AID-RNAi screening approaches. Here we discuss the function of the suppressor genes briefly and propose a hypothetical model for the LBS pathway in *C. elegans* (Figure 3).

### Regulation of LBS by IRE-1 and XBP-1

We unbiasedly detected IRE-1, which was previously proposed as a sensor for LBS in yeast and *C. elegans*, and its target *xbp-1* (Thibault *et al.* 2012). This confirms the selectivity of our screen. Unfolded proteins in the ER lead to IRE1 oligomerization and the subsequent stimulation of its endoribonuclease activity and splicing of the transcription factor *xbp-1*. However, monomeric IRE1 still displays RNase activity and splices XBP1 mRNA in HeLa cells during LBS (Kitai *et al.* 2013; Ho *et al.* 2020). Thus, we confirmed that IRE-1 branch acts as a major sensor of LBS.

### Immunity response network regulates lipid bilayer stress

Knocking down phosphatidylcholine synthesis leads to the activation of genes involved in the immune response (Koh *et al.* 2018). Many of these transcripts are upregulated in an IRE-1-dependent manner. In addition to *ire-1*, we detected the NRF1,2,3 homolog *skn-1* and the GATA transcription factor *elt-2*. Both are involved in p38-mediated innate immunity (Block *et al.* 2015; Ewald 2018). SKN-1 is a major transcription factor for promoting oxidative stress resistance (Blackwell *et al.* 2015). There are four isoforms of SKN-1: *skn-1a*, *b*, *c*, *d* (Blackwell *et al.* 2015). A previous study revealed that IRE-1 had an additional mode-of-action in its monomeric state: elevated levels of reactive oxygen species leads to sulfenylation of cysteine residues in IRE-1 and activates SKN-1a via the p38 MAPK (Hourihan *et al.* 2016). Isoform *skn-1a* is similar to mammalian NRF1, which regulates proteostasis and is a transmembrane protein located in the ER (Wang and Chan 2006; Glover-Cutter *et al.* 2013; Lehrbach and Ruvkun 2019). Curiously, *mdt-15* and *skn-1c* but not *skn-1a*, co-regulate targets involved in detoxification, such as *gst-4* (Goh *et al.* 2014). This suggests that SKN-1a is activated by loss of *mdt-15* and works independently of MDT-15. Knockdown of *skn-1* does not only block LBS response but also reverses the small body size and the small number of eggs laid (although these eggs do not hatch as *skn-1* knockdown is embryonic lethal; Supplementary Table 1). This suggests that some of the observed phenotypes in *mdt-15* mutants or knockdowns are *skn-1*-dependent. The mammalian SKN-1c ortholog NRF2 has been shown to have protective functions during palmitate-induced lipotoxicity in mammalian cells (Cunha *et al.* 2016; Park *et al.* 2015). Taken together, this implies a potential isoform-specific role for *skn-1* during LBS.

The GATA transcription factor *elt-2* is essential for the mesodermal cell fate and development of the intestine. While the null mutation of *elt-2* is embryonic lethal, post-developmental knockdown shortens lifespan, and overexpression extend lifespan (Mann *et al.* 2016). We observed developmental arrest after *elt-2* knockdown. These arrested larvae remained susceptible to heat and tunicamycin treatments, indicating that the UPR was still intact. Like *skn-1*, *elt-2* is recruited to promoters during *Pseudomonas aeruginosa* infection and co-regulates targets in a p38-mediated fashion (Block *et al.* 2015). Furthermore, *elt-2* and *mdt-15* cooperate during heavy metal intoxication (Shomer *et al.* 2019), supporting the idea of a transcription factor network that cooperatively regulates different stress responses.

### Modulators and activators of the LBS response (let-607, gsk-3, and drl-1)

We found three genes, *let-607*, *gsk-3*, and *drl-1*, that are implicated in modulating ER stress responses. RNAi of *let-607* suppressed the activation of the *atf-4* reporter (Figure 2c). *let-607*, together with *crh-1* and *crh-2*, was one of the CREB3 orthologs in *C. elegans*. The mammalian Creb3 family consists of five members (CREB3/Luman, CREB3L1/OASIS, CREB3L2/BBF2H7, CREB3L3/CREBH, and CREB3L4) and is related to ATF6 and SREBP (Sampieri *et al.* 2019). All are localized in the ER and, like ATF6, are activated by anterograde transport to the Golgi and subsequent cleavage by S1P or S2P. In humans and mice, CREB3L2 upregulates SEC23 and controls secretion load, especially during bone formation (Saito *et al.* 2009; Tomoishi *et al.* 2017; Al-Maskari *et al.* 2018). CREB3 and CREB3L3 are induced after palmitate-induced ER stress, and knockdown of CREB3 by siRNA sensitizes human islet cells to palmitate-induced ER stress (Cnop *et al.* 2014). CREB3 has been identified in regulating Golgi-stress and activation of ARF4 (Reiling *et al.* 2013). A previous study in *C. elegans* links *let-607* with the upregulation of *sec-23* and other proteins involved in secretion (Weicksel *et al.* 2016). The *let-607* gene has also been identified in a screen for suppressors of PolyQ aggregation and suppresses motility defects caused by mutations in the paramyosin ortholog UNC-15, the basement-membrane protein perlecan UNC-52, the myosin-assembly protein UNC-45, and the myosin heavy chain UNC-54 (Silva *et al.* 2011). In addition, knockdown of *let-607* increased the expression of cytosolic heat-shock proteins. Based on these previous observations and our results, we propose that *let-607*/CREB3 family is sensing LBS and acts together with the other identified transcription factor encoding genes *xbp-1*, *skn-1*, and *elt-2* to mount a unique stress response that is different from the canonical UPR.

*drl-1**/MAP3K3*, also known as *mekk-3*, has been found in a screen for enhancers of dauer formation and extends lifespan by simulating dietary restricted-like conditions (Chamoli *et al.* 2014). Curiously, loss of *drl-1* caused a pale appearance resembling *fat-6*/*7* and *mdt-15* mutants, but the mode-of-action appears to be different. The *drl-1* promoter is expressed in vulval muscles, body wall muscles, hypodermis, seam cells, some neurons, and tissues lining the pharynx and anus, but not the intestine. Additionally, knockdown in the intestine using tissue-specific RNAi did not extend lifespan (Chamoli *et al.* 2014). Knockdown of MDT-15 activates P*atf-4*(uORF)::gfp and P*hsp-4*::*gfp* expression mainly in the intestine (Figure 1b). Therefore, knockdown of *drl-1* acts in a cell non-autonomous manner. *drl-1* decreases fat storage by upregulating fatty acid oxidation (Chamoli *et al.* 2014). The *C. elegans* ortholog of the ribonuclease Regnase-1, *rege-1*, shares many upregulated genes and causes a pale appearance without activation of LBS (Supplementary Table 1; (Habacher *et al.* 2016)). This suggests a link between *drl-1* and *rege-1*. However, knockdown of *rege-1* does not phenocopy loss of *drl-1* (Supplementary Table 1). Despite the striking similarities shared by *rege-1* and *drl-1*, only *drl-1* modulates LBS. Intriguingly, *drl-1* knockdown itself causes ER stress at the L2 stage, and this mounts a protective effect throughout life (Matai *et al.* 2019). Since *drl-1* rewires metabolism by mimicking dietary restriction, we speculate that activation of fatty acid oxidation protects from lipotoxicity. Indeed, during our screen, we observed that starved *mdt-15*::*degron*; TIR1; P*atf-4*(uORF)::gfp transgenic animals in the 24-wells without food failed to upregulate the reporter.

Glycogen synthase kinase-3 (*gsk-3*) has been described as the busiest of all kinases with over 100 targets known and was found to attenuate palmitate-induced apoptosis (Ibrahim *et al.* 2011; Beurel *et al.* 2015). Paradoxically, *gsk-3* inhibits *skn-1* and stabilizes CREB3, two mode-of-actions contradicting the results of our screen (An *et al.* 2005; Barbosa *et al.* 2013). The inhibition of *gsk-3* here does not act as previously reported; therefore, we hypothesize other modes of action. One alternative mechanism could be via autophagy. Activation of the lipid bilayer stress activates autophagy via the IRE-1/XBP-1 axis (Ho *et al.* 2020). Blocking autophagy in this context causes sickness, sterility, and developmental defects. Intriguingly, GSK3 inhibition activates autophagy (Parr *et al.* 2012). We speculate that prior knockdown of GSK3 leads to an elevated rate of autophagy, which protects from LBS and ameliorates the stress response.

### General players in gene expression, but specific for LBS

The last three hits consisted of *gtf-2f2*, *ntl-4*, and *rpl-14*, which are involved in transcription, RNA processing, and translation, respectively. Interestingly, although RNAi against *gtf-2f2*, *ntl-4*, and *rpl-14* inactivate general processes, the heat- or tunicamycin induced UPR remained functioning and was not affected. This favors the model that UPR and LBS are differentially regulated (Figure 3).

### Summary

We demonstrated the feasibility of combining AID and RNAi-based genetic screens. We report the identification of eight novel regulators of the lipid bilayer stress response and grouped them into three categories (Figure 3). *skn-1* and *elt-2*, together with the previously characterized *ire-1*, are transcription factors involved in immune responses. *let-607* may be activated in parallel with the canonical UPR arms, and *drl-1* and *gsk-3* modulate the ER stress response in our suggested model upstream or downstream, respectively. The last category consists of genes involved in general processes of gene expression. Interestingly, all eight novel candidate genes are well-conserved, suggesting the potential implications of these genes in the mammalian lipid bilayer stress response.
